# Metamaterials from Plasma-Treated Block Copolymer Monolith

**DOI:** 10.1021/acsami.5c25786

**Published:** 2026-03-23

**Authors:** Chien Chen, Jui-Chang Chuang, Ke-Hsin Yin, Cheng-Hsun Tung, Kai-Cheng Yang, Yu-Chueh Hung, Chang-Chun Lee, Rong-Ming Ho

**Affiliations:** † Department of Chemical Engineering, 34881 National Tsing Hua University, No. 101, Section 2, Kuang-Fu Road, Hsinchu, Taiwan 30013, R.O.C; ‡ Department of Power Mechanical Engineering, 34881 National Tsing Hua University, No. 101, Section 2, Kuang-Fu Road, Hsinchu, Taiwan 30013, R.O.C; § Institute of Photonics Technologies, 34881 National Tsing Hua University, No. 101, Section 2, Kuang-Fu Road, Hsinchu, Taiwan 30013, R.O.C

**Keywords:** block copolymer, high transmittance, energy dissipation, optical metamaterials, mechanical metamaterials

## Abstract

This work demonstrates a facile method for fabricating thin-film metamaterials with high transmittance and superior impact resistance directly from plasma-treated polystyrene-*block*-polydimethylsiloxane (PS-*b*-PDMS) monoliths. Upon oxygen plasma treatment, the cocontinuous PDMS within the PS matrix is oxidized to SiO_2_, whereas the PS matrix simultaneously decomposes, yielding a nanoporous SiO_2_ thin film with a well-ordered network texture. The resulting monolith exhibits an ultralow refractive index and excellent optical transmittance, thereby functioning effectively as an optical metamaterial. Concurrently, its deliberate nanonetwork architecture affords exceptional impact resistance, highlighting its characteristics as a mechanical metamaterial. As a proof of concept, the SiO_2_ nanonetwork is applied as a protective overlayer for indium tin oxide (ITO) conductive lines, demonstrating its potential as a ductile glass coating for fragile conductive components in semiconductor devices.

## Graphical abstract

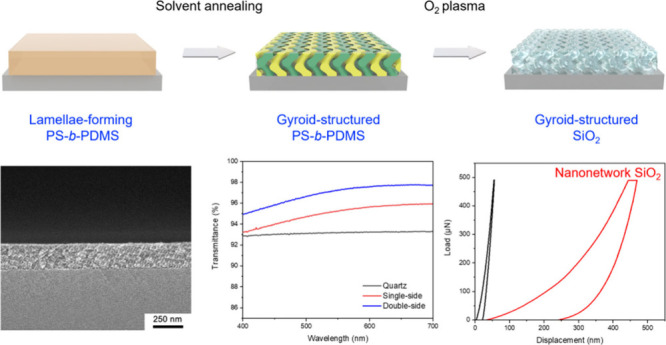

## 1 Introduction

Glass, a ubiquitous material for optics, has been extensively utilized in optical applications due to its excellent properties such as high transmittance, thermal stability, and chemical durability.^1^ However, the intrinsic brittleness of glass limits its utility in scenarios that require flexibility, high-impact resistance, and durability under stress. To address this challenge, developing glass materials with enhanced mechanical properties has drawn considerable attention but remains a significant challenge in materials science. As proposed by Pendry et al.,^2,3^ metamaterials derive their effective properties not from the intrinsic behavior of their constituent bulk materials, but rather from their deliberately engineered structures. While metamaterials were originally investigated for electromagnetic properties, the concept has since expanded across diverse domains, including acoustics, thermodynamics, optics, and mechanics. Within this framework, optical metamaterials exploit nanoscale architectures to realize unusual refractive indices and enhanced light transmission,^4^ while mechanical metamaterials exhibit unconventional responses, including negative Poisson’s ratio, tunable stiffness, and enhanced toughness through structural designs. Recent advances demonstrate that three-dimensional architected networks can significantly enhance impact tolerance and energy dissipation by promoting geometry-governed deformation mechanisms, even in materials that are intrinsically brittle.^5–7^ These studies establish a design paradigm in which structural topology dictates mechanical performance, enabling the transformation of fragile solids into mechanically resilient metamaterials.

To exploit the mechanical advantages imparted by topological design, it is essential to construct ordered three-dimensional (3D) network architectures at the nanoscale. While additive manufacturing such as interference lithography,^8^ projection microstereolithography,^9,10^ photolithography,^6^ and 3D printing^5,11,12^ enable the creation of 3D scaffolds for mechanical metamaterials; achieving well-defined nanoobjects at sub-100 nm length scales remains highly challenging for those top-down approaches. These methods typically rely on serial or multistep processing, require sophisticated instrumentation, and involve high capital and operational costs, which collectively limit their scalability for large-area or high-throughput fabrication. Even with recent advances pushing the resolution to 400–500 nm, further miniaturization is still limited. Alternatively, the self-assembly of block copolymers (BCPs) offers a scalable bottom-up route for fabricating well-ordered nanonetwork architectures with structural periodicities below 100 nm. In contrast to conventional top-down approaches, BCP self-assembly enables parallel, large-area fabrication with reduced processing complexity and cost.^13–16^ Through spontaneous microphase separation, BCPs can self-assemble into periodic morphologies, particularly intricate well-ordered nanonetworks such as double gyroid (DG, *Ia3̅d*)^17–20^ and double diamond (DD, *Pn3̅m*)^21–23^ with highly tunable and precise dimensions determined by their molecular weight and composition. This self-organizing capability opens a pathway to scalable fabrication of architected materials in the nanoscale regime.

Beyond their roles as self-assembled structures, those nanonetworks can be transformed into functional materials through templating techniques.^24^ A porous template with interconnected nanochannels can be obtained through selective removal of one of the constituent blocks in BCPs via hydrolysis,^25,26^ acid etching,^27^ ozonolysis,^28^ and UV irradiation.^29^ By using the fabricated nanoporous polymers as templates, a variety of functional hybrids or porous materials can be obtained through templated syntheses such as templated polymerization,^30,31^ sol–gel reaction,^32–36^ electrochemical deposition,^37–39^ electroless plating,^40,41^ and atomic layer deposition (ALD).^42–44^ Previous studies have demonstrated the effectiveness of BCP templated synthesis for fabricating nanonetwork structures with controlled topology at nanoscale dimensions to enhance mechanical performance. Ho et al. demonstrated that nanonetwork epoxy resins with gyroid and diamond architectures exhibited improved energy dissipation under mechanical loading.^45^ Furthermore, diamond-structured calcite single crystals, inspired by starfish skeletons, showed exceptional strength and energy absorption due to uniform stress distribution and pronounced nanoscale effects.^35^ Similarly, nanonetwork hydroxyapatite, mimicking mantis shrimp structures, showed enhanced energy dissipation and mechanical toughness, attributed to the periodic architecture and characteristic nanoscale features.^46^ Although nanonetwork silica structures with high optical transmittance have been realized via templated sol–gel synthesis,^32^ those processes often require multiple steps and long processing times. In addition, their mechanical properties remain insufficiently characterized. Other template-based strategies have also been reported for transparent or hybrid architectures; however, these approaches remain challenging to exploit for the integration of nanoscale mechanical robustness in thin-film systems.^47,48^ In contrast, the present method enables direct conversion of self-assembled PS-*b*-PDMS thin films into nanonetwork SiO_2_ through a single-step oxygen plasma treatment, eliminating wet etching and thus significantly simplifying the fabrication process. These limitations highlight the need for a more efficient strategy that leverages structural design to overcome the intrinsic brittleness of glass and unlock its full mechanical and optical potential.

Constructing nanonetwork architecture as monolithic thin films represents a viable and scalable strategy for fabricating mechanical metamaterials for applications in devices compatible with micro/nanoelectromechanical systems (MEMS/NEMS) processes. Solvent vapor annealing (SVA) has emerged as an effective approach for directing the self-assembly of BCPs into well-ordered thin films.^36,49–57^ By exposing BCP films to carefully selected solvent vapors, the effective glass transition temperature (*T*_g_) of the BCP is significantly reduced due to the plasticization effect (i.e., the enhanced mobility of polymer chains), allowing thermodynamically stable or metastable self-assembled morphologies to form in BCP thin films through microphase separation. Notably, SVA has been successfully used to achieve thin films with double gyroid (DG) and double diamond (DD) morphologies, demonstrating the effectiveness of this methodology in producing network-structured thin films.

Herein, by taking advantage of the easy formation of nanonetwork-structured thin films from BCP self-assembly via SVA process, this work aims to demonstrate a streamlined and template-free approach for fabricating ductile glass monolith with high transmittance. Figure 1 shows the schematic illustration for the fabrication of the glass film simply from oxygen plasma-treated polystyrene-*block*-polydimethylsiloxane (PS-*b*-PDMS) thin film. A lamellae-forming PS-*b*-PDMS sample is uniformly deposited onto a quartz substrate, resulting in a uniform thin film (Figure 1a). By taking advantage of solvent annealing with a PS-selective solvent, chlorobenzene, a gyroid-structured PS-*b*-PDMS monolith can be fabricated via controlled self-assembly (Figure 1b). This controlled self-assembly is achieved by selectively swelling the polystyrene block, thereby fine-tuning the volume fraction of the BCP to induce a phase transition from lamellae to gyroid.^58,59^ In contrast to the precise synthesis of BCPs with compositions inherently located within the gyroid region, this method offers a simpler and more scalable route for production. Subsequently, oxygen plasma treatment can be applied to the thin film to convert the PDMS into SiO_2_ and to decompose the PS simultaneously, giving a well-ordered nanonetwork glass monolith (Figure 1c) while eliminating the need for templating strategies extensively studied for diverse material applications.

**Figure 1:**
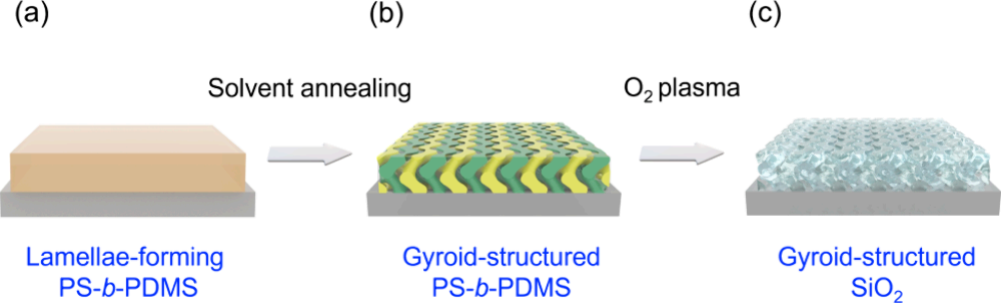
Schematic illustration for fabrication of gyroid-structured SiO_2_ monoliths. (a) PS-*b*-PDMS thin film after spin coating or dip coating. (b) Gyroid-structured PS-*b*-PDMS thin film after solvent annealing using a PS selective solvent. (c) Gyroid-structured SiO_2_ thin film after oxygen plasma treatment for conversion of PDMS into SiO_2_ and removal of PS simultaneously.

This method provides a straightforward one-step route for fabricating well-ordered nanonetwork glass monoliths, simplifying the process compared with conventional BCP-templated synthesis. The gyroid-structured SiO_2_ with its open-cell porous architecture at the nanoscale enables the fabrication of monoliths with exceptional mechanical properties due to its deliberate structuring. The continuous and periodic nanonetwork promotes efficient energy dissipation and impact tolerance, thereby enhancing mechanical compliance without compromising structural integrity. In addition to mechanical robustness, the inherently high porosity of the gyroid network contributes to elevated optical transmittance, offering multifunctional potential in applications that demand both mechanical strength and optical transparency. This innovative strategy presents a cost-effective and scalable route for developing mechanically robust glass monolith without compositional modification, providing a promising candidate for the integration of thin-film technology into next-generation devices via MEMS/NEMS-compatible processes.

## 2 Results and Discussion

### 2.1 Fabrication of Gyroid-Structured PS-*b*-PDMS Monoliths

A single composition PS-*b*-PDMS (*f*_*PDMS*_^*v*^ = 0.42, *M*_*n*_^*PS*^ = 12,400 g mol^–1^, *M*_*n*_^*PDMS*^ = 10,800 g mol^–1^, *Đ* = 1.06) was synthesized and used in this work (see Supporting Information for synthetic details and corresponding characterization). PS-*b*-PDMS was selected due to its high interaction parameter, enabling reliable self-assembly at relatively low molecular weights and access to small feature sizes.^60^ The intrinsic self-assembled morphology of PS-*b*-PDMS prepared by solution casting using a neutral solvent (cyclohexane) followed by thermal annealing at 180 °C for 72 h can be identified as a lamellar morphology based on small-angle X-ray scattering (SAXS) and transmission electron microscopy (TEM) results (Figure S1). By taking advantage of controlled self-assembly using a PS-selective solvent (chlorobenzene) for solution casting, a morphological evolution from the thermodynamically stable lamellar phase to a metastable double gyroid (DG, *Ia3̅d*) phase can be observed by TEM (Figure S2a), and further verified by SAXS with the relative *q* values at √6:√8:√14:√16:√20:√24:√30:√38:√42:√50:√54 (Figure S2b).^58^ To fabricate gyroid-structured PS-*b*-PDMS monoliths, thin films with different thicknesses were prepared by spin coating or dip coating onto quartz substrates. Solvent vapor annealing (SVA) was performed in a custom-built sealed chamber using chlorobenzene vapor under controlled flow conditions (Figure S3), and the film swelling behavior during annealing was monitored (Figure S4). Under these conditions, well-ordered gyroid-structured PS-*b*-PDMS films were reproducibly obtained. Figure 2a shows the atomic force microscopy (AFM) height image of the PS-*b*-PDMS thin film after solvent annealing followed by reactive ion etching (RIE) treatment to reveal the topographic texture, in which a characteristic double wave pattern can be clearly identified, suggesting the formation of gyroid-structured monolith with the (211) plane on the surface. Note that there is a thin PDMS wetting layer due to the low surface tension of PDMS that can be easily removed by CF_4_/O_2_ RIE treatment. A top-view field-emission scanning electron microscopy (FESEM) image (Figure 2b) further confirms the formation of the monolith with a network morphology. To further examine the self-assembled morphology, grazing-incidence small-angle X-ray scattering (GISAXS) experiment was conducted on the solvent-annealed sample without RIE treatment; as shown in Figure 2c, Bragg diffraction peaks of (211) and (220) can be clearly identified at which the rod-like diffraction patterns suggest the formation of a monolith with a preferential (211) orientation of the gyroid phase. Most importantly, the results clearly confirm the formation of gyroid-structured monolith regardless of the PDMS wetting layer. Notably, GISAXS measurements were performed at various annealing times (Figure S5); the experimental results reveal that the ordering of the forming gyroid phase progressively increases with the annealing time; after 2 h annealing, a well-defined gyroid-structured monolith can be successfully fabricated.

**Figure 2:**
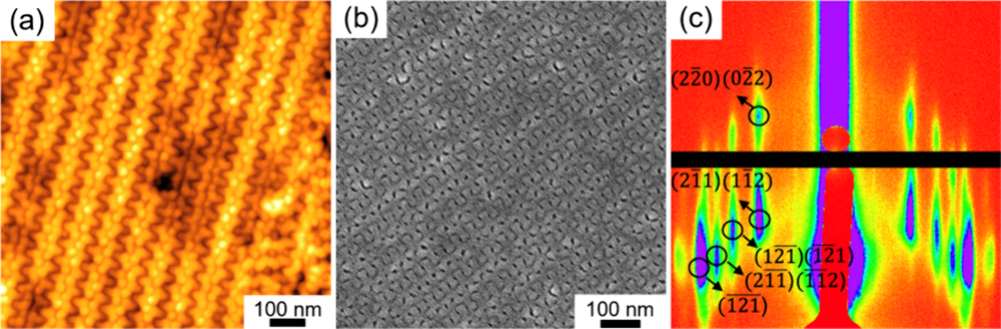
Characterization of the PS-*b*-PDMS film. (a) AFM height image. (b) Top-view FESEM micrograph after RIE treatment. (c) GISAXS pattern of the solvent-annealed PS-*b*-PDMS film.

### 2.2 Oxygen Plasma Treatment of Gyroid-Structured PS-*b*-PDMS Monoliths

Following the fabrication of the gyroid-structured PS-*b*-PDMS thin film, oxygen plasma treatment was applied to selectively oxidize the PDMS into SiO_2_ while simultaneously decomposing the PS matrix, yielding a well-ordered gyroid-structured SiO_2_. Prior to this conversion, a thin PDMS wetting layer formed on the film surface, attributed to the inherently low surface tension of PDMS. To remove this layer and ensure a clean surface for subsequent plasma processing, a brief RIE treatment using a CF_4_/O_2_ gas mixture was performed. Figure S6 presents top-view FESEM images of the gyroid-structured film before and after CF_4_/O_2_ RIE treatment (Figure S6a) at which the underlying nanoscale pattern is obscured by the PDMS wetting layer. By contrast, after RIE treatment, the removal of this layer exposes the clear morphology of the PS-*b*-PDMS gyroid structure (Figure S6b) fabricated, confirming the effectiveness of the wetting layer removal process. To investigate the etching behavior of PS matrix and the conversion of PDMS gyroid microdomain, both oxygen plasma cleaner (isotropic etching) and O_2_ reactive ion etching (anisotropic etching) were employed. The plasma cleaner leads to uniform etching in all directions driven primarily by chemically reactive neutral species, while RIE introduces ion bombardment under an electric field, resulting in vertically biased etching. These intrinsic differences significantly influence the preservation or deformation of the 3D gyroid morphology during the etching process. Note that the etching behavior within nanonetwork structures fundamentally differs from that of conventional vertical structures commonly targeted in plasma etching processes. Vertical etching typically relies on anisotropic ion bombardment, enabled by high plasma power and low working pressure, which increases the mean free path (λ) of reactive species. The mean free path is defined by Equation 1:

$$\lambda =\frac{kT}{\sqrt{2}\pi d^{2}P}$$ 1

where *k* is the Boltzmann constant, *T* is the temperature, *d* is the molecular diameter, and *P* is the working pressure. In contrast to the relatively simple geometries of cylinder and lamellar morphologies, gyroid nanonetworks with densely interconnected three-dimensional frameworks impose substantial challenges on anisotropic etching processes due to their complex topology and spatial continuity. Owing to their complex 3D-interconnected structure, certain regions within gyroid networks inevitably shield other areas, making those regions inaccessible to reactive species. This phenomenon can be demonstrated in Figure S7, where RIE conducted at 100 W for 60 min cannot fully remove the PS matrix, resulting in incomplete etching and residual polymer content within the structure.

In contrast, the oxygen plasma cleaner achieves a more isotropic etching environment due to design features such as higher operating pressure (i.e., a shorter mean free path) and downstream plasma generation, resulting in predominantly chemical reactions without directional ion bombardment.^61,62^ Those designs facilitate the diffusion of reactive oxygen species throughout the porous nanonetwork. The shorter mean free path leads to more frequent gas-phase collisions, which suppress directional transport and reduce direct collisions with the structure, allowing reactive species to penetrate confined regions of the nanonetwork more effectively. As a result, oxygen plasma cleaner enables deeper and more uniform etching, making it particularly well-suited for fabricating thick, free-standing gyroid-structured SiO_2_ monoliths while preserving the nanonetwork structure. The uniformity and structural integrity of the gyroid-structured SiO_2_ are critically governed by plasma chemistry, along with key processing parameters including plasma power, etchant gas flow rate, and treatment duration. These factors collectively regulate the oxidation efficiency of PDMS and the simultaneous removal of the PS matrix. Among them, plasma power plays a particularly critical role because it directly controls ion energy through RF power and self-bias. Excessive radio frequency (RF) power has been shown to substantially increase ion energy, which can induce nonuniform etching behavior and damage delicate nanonetwork architectures.^63^

Importantly, the conversion process must maintain a balance between PDMS oxidation and PS decomposition to avoid both under- and overetching. Rapid removal of the PS matrix can lead to structural collapse of the nanonetwork as unoxidized PDMS remain in a viscoelastic state at room temperature, undermining the framework before sufficient oxidation into rigid SiO_2_ can be achieved. Conversely, insufficient oxidation promotes the formation of undesirable byproducts such as silicon oxycarbide (SiOC), which arises from partial conversion of PDMS under suboptimal plasma conditions. Therefore, meticulous control and optimization of process parameters are imperative to achieve complete oxidation throughout the entire film thickness while maintaining the structural integrity of the delicate nanonetwork structure.

Figure 3 presents cross-sectional SEM micrographs, demonstrating the influence of specific plasma parameters on the conversion process. Under plasma conditions of 100 W power, 10 sccm flow rate, and a 15 min treatment duration, both thinner (Figure 3a) and thicker (Figure 3b) PS-*b*-PDMS films exhibit limited oxidation with only an etched layer of approximately 45 nm on the top surface. As shown in Figure 3c, extending the treatment time to 60 min increases the conversion depth to approximately 110 nm in a thinner film. A similar result can be observed in a thicker film (Figure 3d), where the same plasma conditions also yield a conversion depth of 110 nm, demonstrating that prolonged treatment is effective across different initial thicknesses. This improvement is attributed to the extended exposure time, which allows reactive oxygen species to diffuse deeper into the gyroid nanonetwork and fully oxidize the PDMS phase into SiO_2_. Nevertheless, prolonged plasma exposure beyond the optimized condition can lead to gradual thinning of the converted SiO_2_ framework. As shown in Figure S8, an additional oxygen plasma treatment at 100 W for 60 min results in a noticeable reduction in film thickness, indicating slow etching of amorphous SiO_2_. This observation highlights the importance of defining a controlled processing window to achieve complete conversion while minimizing undesired SiO_2_ loss. To evaluate the influence of oxygen flow rate on oxidation depth, two samples with comparable initial film thicknesses were prepared and treated under identical plasma conditions (100 W, 60 min), with the oxygen flow rate as the sole variable. As shown in Figure 3c, using a flow rate of 10 sccm results in an oxidation depth of approximately 110 nm. In contrast, increasing the flow rate to 20 sccm (Figure 3e) extends the conversion depth to approximately 180 nm, demonstrating that a higher oxygen supply significantly improves oxidation efficiency. Higher flow rates increase the concentration of reactive oxygen species (ROS) and enhance gas transport, ensuring a steady supply of oxidizing agents throughout the nanonetwork.^64^ These combined factors promote deeper and more uniform oxidation, overcoming the diffusional limits present at lower flow rates. These findings confirm that maintaining moderate plasma power combined with optimized oxygen flow rate and treatment duration enables uniform PDMS oxidation while preserving the integrity of the gyroid nanonetwork. This balance between oxidation efficiency and structural stability ensures effective conversion within the porous structure, allowing for optimization of the plasma conditions for controlled oxidation without compromising the nanonetwork morphology. Through meticulous control and optimization of processing parameters, SiO_2_ nanonetworks were fabricated without incomplete conversion to undesired byproducts such as SiOC.

**Figure 3:**
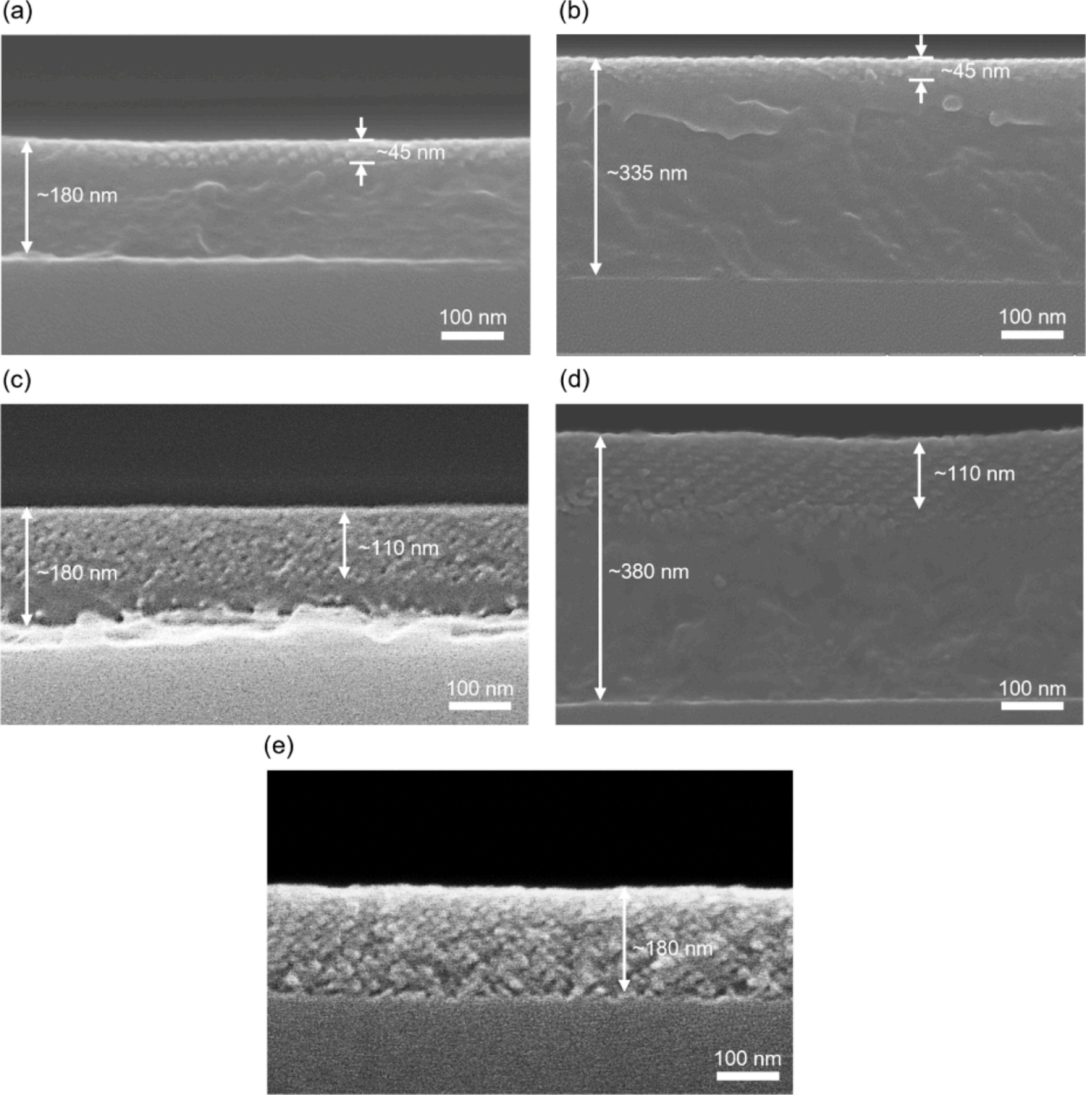
Cross-sectional SEM images of samples treated with oxygen plasma under varying power, flow rate, and processing time, highlighting the conversion of PDMS into SiO_2_. (a, b) 100 W, 10 sccm, 15 min, with different original thicknesses. (c, d) 100 W, 10 sccm, 60 min, with different original thicknesses. (e) 100 W, 20 sccm, 60 min, highlighting the influence of flow rate on the conversion process.

Figure 4a presents the top-view FESEM micrograph of the monolith fabricated after 60 min of oxygen plasma treatment where the (211) pattern is distinctly visible, confirming the preservation of the gyroid morphology. Figure 4b shows the cross-sectional FESEM micrograph, demonstrating the successful fabrication of a gyroid-structured SiO_2_ thin film with a uniform thickness of 200 nm, highlighting the structural integrity and uniformity of the film.

**Figure 4:**
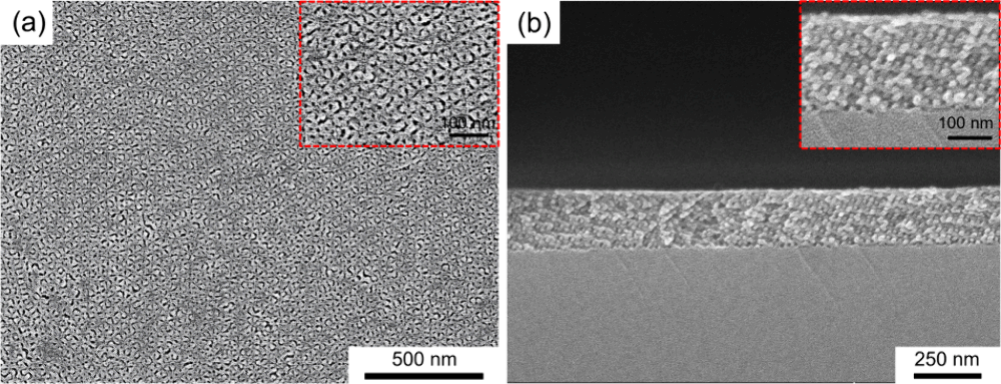
Morphology of gyroid-structured SiO_2_ thin film. (a) Top view. (b) Cross-sectional FESEM micrographs of gyroid-structured SiO_2_ thin film after oxygen plasma treatment for 60 min, converting the PDMS into SiO_2_ and decomposing the PS simultaneously. Insets show magnified views of the gyroid-structured SiO_2_.

### 2.3 Structural and Chemical Characterization of Plasma-Treated Gyroid-Structured SiO_2_

To further investigate the structural evolution of the PS-*b*-PDMS thin film before and after oxygen plasma treatment, 2D GISAXS experiments were conducted. Compared to the untreated sample (Figure S9a), additional reflection corresponding to the (200) plane can be found, indicating the occurrence of structural deformation in the gyroid network after oxygen plasma treatment (Figure S9b). In addition, an additional reflection corresponding to the (110) plane can be observed, suggesting the formation of single-gyroid-like diffraction.^65^ In contrast to the formation of double gyroid phase from self-assembly, these results further indicate that the SiO_2_ gyroid network becomes randomly shifted after conversion of PDMS into SiO_2_ and removal of the PS matrix by plasma treatment. The observed symmetry breaking is not detrimental; instead, it signifies a rearrangement that could enhance the optical properties of thin films. For instance, this symmetry breaking has the potential to open a photonic band gap, creating conditions favorable for the formation of Weyl points; which is crucial for topological photonic applications. The GISAXS signal contrast is significantly enhanced after the plasma treatment. Initially, the contrast originates from the electron density difference between PS and PDMS. After plasma treatment, the contrast is defined by the difference between SiO_2_ and air which substantially alters the scattering contrast. This enhancement in contrast after plasma treatment not only confirms the successful conversion and removal of materials but also improves the clarity and definition of the gyroid nanostructure in the GISAXS patterns.

To characterize the chemical nature of the PS-*b*-PDMS after oxygen plasma treatment, X-ray photoelectron spectroscopy (XPS) was used to investigate the conversion of the PDMS block and removal of the PS block. Figure S10a shows the Si 2p spectra of the thin film before and after oxygen plasma treatment, along with a pure SiO_2_ reference for comparison. The untreated PS-*b*-PDMS sample exhibits a characteristic Si 2p peak at approximately 101.0 eV which corresponds to the silicon oxide backbones in the PDMS block. After oxygen plasma treatment, this peak shifts to a higher binding energy of approximately 103.0 eV, as shown by the blue curve. This shift toward higher binding energies is indicative of the oxidation of PDMS to SiO_2_; the increased electron binding energy reflects the incorporation of oxygen into the silicon atoms, forming a silicon dioxide structure. This result demonstrates the successful conversion of the PDMS block into SiO_2_, in line with the expected chemical transformation. For XPS depth profiling, the PS-*b*-PDMS film was prepared on a Si wafer to enable analysis of the interfacial region of the nanonetwork structure. To investigate the chemical composition of the lower region of the nanonetwork structure, the upper portion of the film was selectively removed using CF_4_/O_2_ RIE. This treatment exposed approximately 40 nm of material adjacent to the Si substrate, isolating the region of interest for chemical analysis. As shown in Figure S10b, the XPS analysis reveals a dominant presence of silicon and oxygen, with the silicon-to-oxygen atomic ratio consistently close to 1:2, indicating complete oxidation of the PDMS framework into SiO_2_. Importantly, no detectable carbon signal was observed throughout the analyzed depth, suggesting the absence of residual PS or partially oxidized species such as SiOC. These results confirm the successful conversion of a chemically uniform SiO_2_ composition within the examined region of the nanonetwork structure.

### 2.4 Gyroid-Structured SiO_2_ Monoliths with Ductility

To demonstrate the targeted ductility of gyroid-structured SiO_2_ monoliths with the character as mechanical metamaterials, nanoindentation tests were employed to provide a comparison with the energy absorption capability of a standard quartz substrate. At an equivalent maximum applied force of 500 μN, the gyroid-structured SiO_2_ exhibits a significant displacement of 469.2 nm, indicating superior deformation performance compared with the quartz substrate, which shows a displacement of 56.3 nm (Figure 5a). Notably, the permanent displacement of gyroid-structured SiO_2_ after loading is more than 8 times that of the quartz substrate. This substantial increase highlights the significant impact of deliberate structuring on the mechanical properties. The mechanical response is highly reproducible, as demonstrated by multicycle nanoindentation tests conducted under different peak loads (150, 300, and 500 μN), which exhibit consistent load–displacement behavior across a range of deformation levels (Figure S11). The architected nanonetwork structure markedly enhances energy dissipation capacity by promoting distributed deformation mechanisms. Moreover, the energy dissipation index was employed to quantitatively estimate the energy dissipation capability by representing the dissipated energy (*W*_*p*_) from plastic deformation relative to the total contact energy (*W*_*t*_); the index describes the relationship between the dissipated energy from plastic deformation and the elastic store energy (*W*_*e*_). Mathematically, it is expressed by the following Equation 2:

$$\mathrm{Energy}\;\mathrm{dissipation}\;\mathrm{index}=\frac{W_{p}}{W_{t}}=\frac{W_{p}}{W_{p}+W_{e}}$$ 2

where *W*_*p*_ and *W*_*e*_ indicate the dissipated energy associated with plastic deformation and elastic deformation, respectively. *W*_*t*_ denotes total contact energy. The plastic dissipation energy of the quartz substrate was determined from the gray area shown in Figure 5b, with a calculated value of 2.3 pJ. The total contact energy is analyzed and found to be 9.3 pJ. Accordingly, the energy dissipation index for the quartz substrate was computed to be 24.9%. By comparison, the plastic dissipation energy of gyroid-structured SiO_2_ was determined from the red area in Figure 5b, yielding a calculated value of 33.6 pJ. The total contact energy was analyzed and reported as 64.0 pJ, leading to an energy dissipation index of 52.5%.

**Figure 5:**
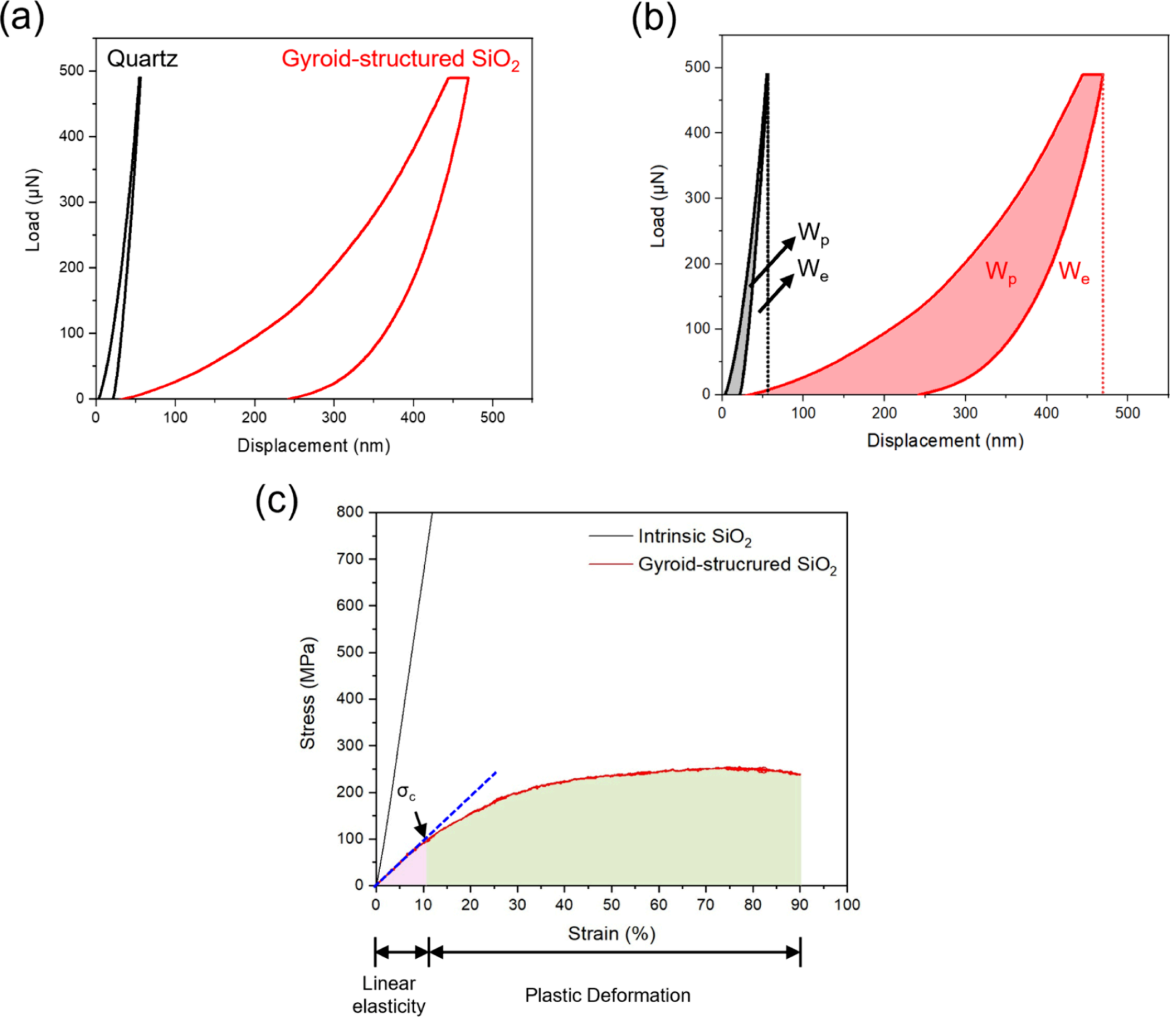
Mechanical properties of gyroid-structured SiO_2_ thin films. (a) Load–displacement curves for the nanoindentation test of quartz and gyroid-structured SiO_2_ films. (b) Elastic energy (*W*_*e*_) and plastic energy (*W*_*p*_) dissipation during the indentation of the gyroid-structured SiO_2_ thin film. (c) Engineering stress–strain curves obtained from uniaxial microcompression tests comparing intrinsic SiO_2_ and gyroid-structured SiO_2_.

To further investigate the effect of the gyroid architecture on the mechanical properties, pillar-shaped samples of gyroid-structured SiO_2_ with a diameter of approximately 2.5 μm were prepared through focused ion beam (FIB) milling for comparison with intrinsic SiO_2_ through the similar preparation (i.e., sol–gel reaction). Uniaxial compression was applied using a nanoindenter equipped with a flat punch with a diameter of 10 μm to compare the mechanical performance of intrinsic SiO_2_ and gyroid-structured SiO_2_. Figure 5c exhibits representative engineering stress–strain curves for both materials. The intrinsic SiO_2_ (black curve) undergoes elastic deformation and is expected to experience catastrophic fracture without plastic deformation, consistent with its brittle material nature. The Young’s modulus of the intrinsic SiO_2_ was calculated from the slope of the linear elastic region, giving a value of approximately 6.7 GPa. In contrast, gyroid-structured SiO_2_ (red curve) initially follows the linear elastic deformation regime and exhibits a linear stress–strain relationship from 0% to 10% strain with a calculated Young’s modulus of 1.0 GPa. In this elastic deformation region, the deformation is fully recoverable, consistent with Hooke’s law. Beyond the elastic limit, the stress–strain curve transforms into a plastic deformation region, in which the material undergoes permanent deformation. In this region, stress gradually increases as strain progresses. The transition from elastic to plastic deformation is marked by a peak stress of approximately 100 MPa at a strain of 10%. This behavior highlights the ability of gyroid-structured SiO_2_ to sustain strain without fracturing, demonstrating a significant departure from the brittle behavior of intrinsic SiO_2_. As the strain exceeds 10%, the material enters a fully plastic deformation regime, exhibiting both elasticity and ductility, as indicated by its ability to recover part of the deformation during initial loading and its capacity to sustain significant strain without fracturing. This unique combination arises from the gyroid nanonetwork, which effectively redistributes stress along the surface of the struts and thus accommodates deformation over a wide strain range. Stress continues to increase progressively with strain, indicating the capacity to dissipate energy under deformation. At higher strain, beyond 45%, the stress–strain curve reaches a plateau region, in which the stress remains nearly constant despite further significant deformation. In sharp contrast to the brittle fracture observed in intrinsic SiO_2_, this plateau behavior underscores the exceptional ductility and flexibility of gyroid-structured SiO_2_ due to the effect of deliberate structuring on mechanical properties.

Furthermore, nanoscale dynamic mechanical analysis (nanoDMA) was employed to gain insight into the damping behavior for the nanonetwork SiO_2_ compared with intrinsic SiO_2_. Specifically, the study utilized a nanoDMA setup with displacement amplitudes ranging from 18.22 to 18.26 nm, operating at a frequency of 200 Hz. As shown in Figure S12, the results, quantified by a damping factor, tan δ, indicate a significant difference in damping capacity, where intrinsic SiO_2_ displays a low tan δ value of approximately 0.12, as expected for a glass material with low damping, whereas the gyroid-structured SiO_2_ exhibits a tan δ value of approximately 1.07, suggesting a significant improvement in damping performance. Note that the damping angle (δ) is the phase lag between stress and strain in a viscoelastic material during oscillatory deformation. Accordingly, the damping angle of intrinsic SiO_2_ was estimated as 6.8°; by contrast, the gyroid-structured SiO_2_ exhibits a damping angle of approximately 47.0°, indicating a higher damping behavior than that of intrinsic SiO_2_. Those results demonstrate the significant effect of the deliberate structuring as nanonetwork on damping behavior, in line with the results from nanoindentation and compression tests. The enhanced damping capacity of gyroid-structured SiO_2_ underscores its potential for energy dissipation applications, particularly in vibration and shock mitigation.

### 2.5 Demonstration of Gyroid-Structured SiO_2_ for Improved Mechanical Properties

To assess the mechanical performance of the gyroid-structured SiO_2_, an experiment was conducted by placing a SiO_2_ nanonetwork layer on the top surface of an indium tin oxide (ITO) conductive line. The configurations with and without the SiO_2_ nanonetwork were compared to evaluate its protective function through the corresponding changes in electrical resistance under stress. The ITO conductive line, with a width of 0.1 mm, was subjected to electrical resistance measurements via a Keithley instrument. A compressive force was applied to the top surface of the conductive line using an MTS electromechanical test system equipped with a tip diameter of 0.45 mm. The detailed layout of the testing configuration is shown in Figure S13, where one of the parallel ITO lines is selectively covered with a gyroid-structured SiO_2_ thin film. This setup enables a direct comparison of resistance changes under mechanical loading between protected and unprotected ITO lines. Figure 6a shows the relationship between compressive force and electrical resistance over time. Under a compressive force exceeding 9 kgf, the ITO conductive line without the SiO_2_ nanonetwork begins to buckle and crack after a certain duration, resulting in a sharp increase in electrical resistance. In contrast, the electrical resistance of the ITO line protected with the SiO_2_ nanonetwork layer remains stable at 10.7 kΩ ± 3% under similar compression conditions, even when the compressive force exceeds 16.8 kgf. At the initial region, the applied force remains nearly constant. With continuous loading, there is a gradual increase in applied compression force, which then reaches a plateau. After 100 s for loading, the force in the ITO line protected by gyroid-structured SiO_2_ thin film continues to increase smoothly, suggesting the occurrence of gradual plastic deformation due to effective stress redistribution. In contrast, the ITO line without protection from gyroid-structured SiO_2_ approaches its mechanical limit and eventually experiences mechanical failure due to stress concentration in localized areas, leading to microcracks and dislocation pile-up. Accordingly, the nanonetwork structure effectively prevents collapse by redistributing stress. While the unprotected sample is more prone to dramatic mechanical failure with electrical resistance spikes, the ITO line protected by the gyroid-structured SiO_2_ film may undergo strain hardening, allowing it to sustain additional loading and avoid catastrophic failure through effective stress redistribution.

**Figure 6:**
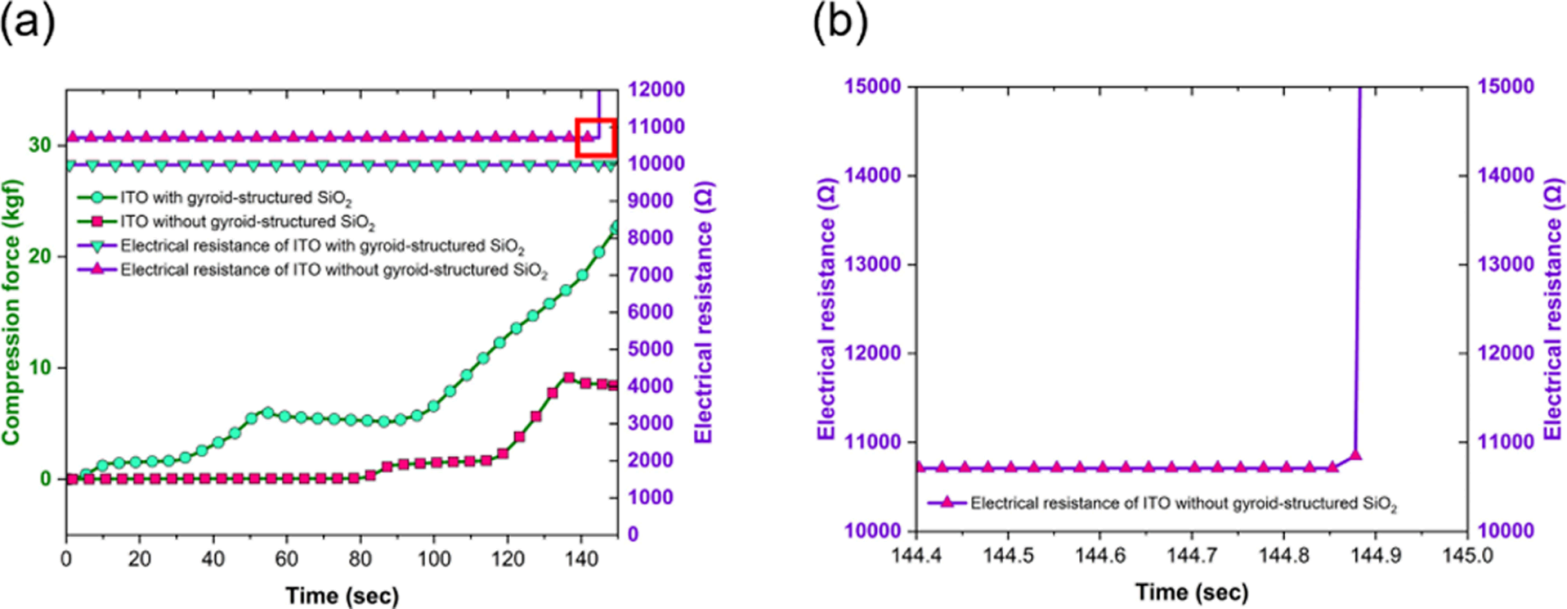
Protective performance of gyroid-structured SiO_2_ films on ITO. (a) Compression force and electrical resistance as a function of time for ITO with and without gyroid-structured SiO_2_ films. (b) Magnified view of the sudden resistance changes in ITO without the gyroid-structured SiO_2_ film, highlighting failure under compression.

Figure 6b provides a magnified view of the resistance change in the unprotected ITO line, emphasizing its mechanical failure. These findings demonstrate that the nanonetwork structure effectively protects the ITO conductive line by preventing mechanical failure, maintaining stable electrical resistance, and providing superior impact resistance.

Owing to the integration of mechanical toughness, energy dissipation capacity, low refractive index and thin-film processability, gyroid-structured SiO_2_ emerges as a highly promising material for advanced electronic and photonic packaging. These properties enable its utilization in systems requiring simultaneous mechanical reliability, optical performance, and miniaturization. In addition to its demonstrated role as a protective overlayer for ITO lines, the gyroid-structured SiO_2_ monolith shows broad applicability across semiconductor technologies. Its 3D nanonetwork architecture facilitates stress redistribution and plastic deformation via strut bending,^66^ making it effective as a stress-buffering interlayer in 2.5D and 3D integrated circuit (IC) packaging to mitigate interfacial delamination and thermomechanical mismatch.^67^ In optical modules such as silicon photonics and vertical-cavity surface-emitting laser (VCSEL) packaging, where waveguides, photodetectors, and modulators are prone to mechanical failure, the gyroid-structured SiO_2_ functions as a transparent mechanical shield. Its nanoscale porosity and well-ordered morphology provide both impact resistance and optical compatibility, preventing crack propagation while minimizing optical loss.^68^ This makes it well suited for photonic-integrated packaging platforms requiring co-optimization of mechanical and optical functions. Its combination of transparency and mechanical resilience also supports its application as a thin-film encapsulation layer for MEMS/NEMS and CMOS image sensors (CIS), where robust protection with minimal optical interference is required. At the same time, the gyroid morphology provides mechanical support and dimensional precision, making it suitable as a scaffold for micro-optical components and as a multifunctional barrier layer in redistribution layers (RDLs) and interposer structures.^69^ These applications are supported by its ductility, low modulus, and high energy dissipation, positioning gyroid-structured SiO_2_ as a versatile platform for next-generation packaging technologies.

### 2.6 Gyroid-Structured SiO_2_ Monolith with High Transmittance

For applications of gyroid-structured SiO_2_ monoliths, it is necessary to consider the variations in the optical performance of the glass because of its excellent intrinsic properties of high transmittance. UV–visible transmittance measurements were conducted over the wavelength range of 400 to 700 nm, corresponding to the visible light region. The film was coated onto a highly transparent substrate (quartz) for the measurements. Figure 7a shows the optical transmittance of quartz substrates coated with single-sided and double-sided gyroid-structured SiO_2_ films. The bare quartz substrate exhibits a consistent transmittance of approximately 93.0%, showcasing its inherent optical clarity and serving as a reference for assessing improvements from subsequent treatments. By contrast, the quartz substrate coated with a gyroid-structured SiO_2_ thin film achieves an average transmittance of approximately 95.2% across the visible spectrum, representing a significant enhancement of 2.2% on average over the untreated quartz. This improvement is attributed to the antireflective properties of the gyroid-structured SiO_2_ thin film on the quartz substrate. The film functions as an effective intermediate layer, minimizing light reflection at the air-quartz interface. This reduction in reflection is primarily attributed to the low refractive index (namely, the high porosity of the gyroid-structured SiO_2_), which is suitable for reducing optical losses and enhancing light transmittance. Note that the glass monolith can also be used as an interlayer material in the MEMS/NEMS fabrication. To justify the optical performance for use as an interlayer, the gyroid-structured SiO_2_ film was applied to both sides of the quartz substrate.

**Figure 7:**
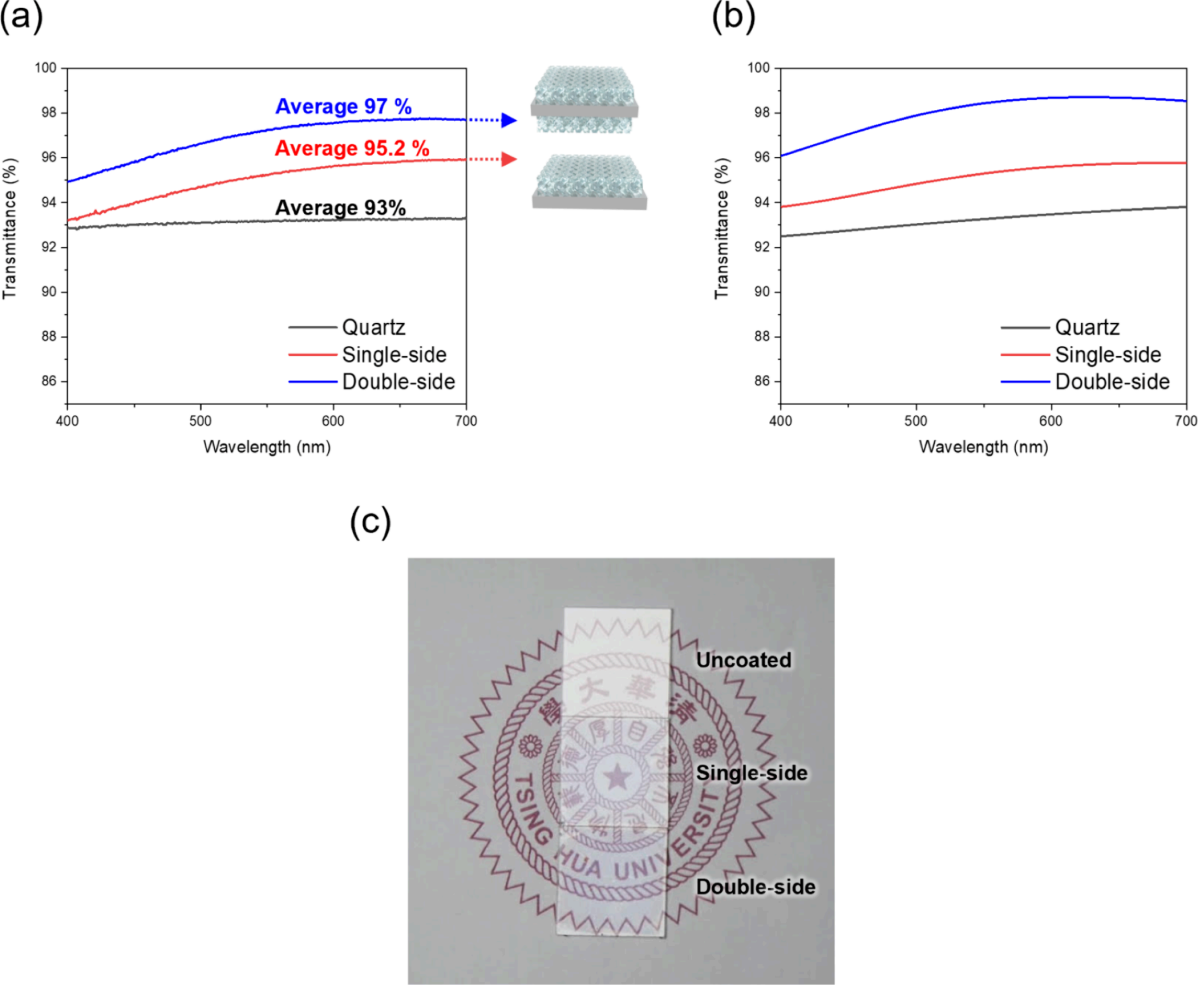
Optical performance of gyroid-structured SiO_2_ thin films. (a) Experimental and (b) simulated optical transmittance spectra of bare quartz, single-side coated, and double-side coated substrates with gyroid-structured SiO_2_ thin films in the visible wavelength range (400–700 nm). (c) Photograph of quartz substrates with and without G-structured SiO_2_ thin-film coating under fluorescent lamp.

This double-sided treatment capitalizes on the ability of the film to reduce reflections not only when light enters the substrate but also as it exits. By mitigating reflections at both interfaces, the double-sided application further minimizes the optical losses, leading to an average transmittance of up to 97.0%. This increment confirms the effectiveness of the double-sided coating strategy in maximizing transmittance and underscores the utility of gyroid structures in advanced optical applications. The simulated results, as shown in Figure 7b, closely align with the experimental findings but with slightly lower transmittance. This discrepancy is likely attributed to fabrication-related defects and nonuniformity in the film deposition process; those are common challenges in the production of highly ordered nanostructured films. The visual effect of the coatings is illustrated in Figure 7c, where a printed pattern beneath the quartz substrates is viewed under a fluorescent lamp. The uncoated substrate shows noticeable reflection and reduced clarity, the single-sided coated substrate displays improved visibility of the underlying pattern, and the double-sided coated substrate offers the clearest and brightest view, directly demonstrating the superior antireflective performance achieved through the double-sided gyroid-structured SiO_2_ coating.

To accurately evaluate the effective refractive index of the gyroid-structured SiO_2_ thin films, the Bruggeman effective medium approximation (BEMA) was employed. This approximation is well-suited to the system studied here, considering the composite nature of the gyroid-structured SiO_2_, composed of both solid SiO_2_ and air voids. The interconnected gyroid morphology forms a heterogeneous medium where the refractive index and porosity jointly influence the optical response. BEMA provides a framework to calculate the effective refractive index (*n*_*eff*_) by accounting for the refractive indices and volume fractions of the constituent materials. The relationship is given by the Equation 3:

$$f\frac{{n_{1}}^{2}-{n_{eff}}^{2}}{{n_{1}}^{2}+2{n_{eff}}^{2}}+\left(1-f\right)\frac{{n_{2}}^{2}-{n_{eff}}^{2}}{{n_{2}}^{2}+2{n_{eff}}^{2}}=0$$ 3

where *n*_1_ and *n*_2_ are the refractive indices of air and SiO_2_, respectively, and *f* is the volume fraction of air voids in the nanoporous gyroid SiO_2_. For this calculation, *f* = 0.66, *n*_1_ = 1 (air), and *n*_2_ = 1.46, the refractive index of dense SiO_2_, determined from optical constants data for SiO_2_ at a wavelength of 550 nm within the visible light spectrum. Incorporating a volume fraction *f* = 0.66 for the air component in the gyroid structure, we calculated the effective refractive index *n*_*eff*_ to be approximately 1.15. This value is significantly lower than that of quartz (*n* = 1.46 at 550 nm), indicating a substantial change in light interaction and thus reducing scattering and reflection at the surface. This leads to more efficient light transmission and enhanced overall transmittance across the visible spectrum.

Moreover, the effect of film thickness on the optical transmittance of gyroid-structured SiO_2_ films was meticulously investigated. As shown in Figure S14a (Supporting Information), with increasing thickness (100, 125, and 150 nm), there is a clear trend of increased transmittance at longer wavelengths. This observation indicates that thicker films are more effective at suppressing reflection in the long-wavelength region, which can be understood in terms of thin-film interference and antireflection behavior approaching the quarter-wave optical thickness (QWOT) condition. Regardless of their thickness, each set of films exhibits a transmittance that exceeds that of the bare quartz substrate, highlighting the effectiveness of the gyroid structure in enhancing optical properties. The films exhibit a broad spectrum of transmittance, similar to that of multilayer antireflection coatings (ARCs), which are generally optimized for specific wavelengths. Unlike traditional ARCs, the gyroid-structured SiO_2_ offers broadband antireflection capabilities. This superior performance is attributed to its unique nanonetwork structure, which not only facilitates multiple internal light interference effects but also acts as a gradient buffer layer, smoothly transitioning the refractive index between air and substrate. This feature is essential for effectively minimizing reflections across a broader range of wavelengths, thus improving the overall optical transmittance of the films. The strong agreement between the experimental data and the simulations (Figure S14b) validates the precision of the simulations in replicating the behavior of the gyroid-structured SiO_2_ films with varying thicknesses. This consistency between experimental and simulation data strongly supports the theoretical models of those advanced materials, affirming their potential to enhance optical properties through strategic adjustments in film thickness and structural configuration.

## 3 Conclusion

This study demonstrates a facile method for fabricating gyroid-structured SiO_2_ monoliths by employing the controlled self-assembly of PS-*b*-PDMS followed by oxygen plasma treatment. The oxygen plasma treatment simultaneously converts PDMS into SiO_2_ and decomposes PS, simplifying and accelerating the fabrication process as compared with traditional templated synthesis methods for the fabrication of well-ordered nanoporous (nanonetwork) SiO_2_ from BCP templates. Balancing PDMS oxidation and PS decomposition requires optimization of plasma parameters, including plasma power, oxygen flow rate, and treatment duration, to ensure uniform conversion and to maintain structural integrity. The combination of topology and nanosized effects in the gyroid-structured SiO_2_ renders it a metamaterial that integrates optical and mechanical functionalities. It exhibits an ultralow refractive index and high transmittance as an optical metamaterial, while nanoindentation tests reveal an energy dissipation index of approximately 52.5%, more than double the 24.9% observed in traditional quartz substrates, and uniaxial compression further confirms superior ductility as a mechanical metamaterial. As a proof of concept, the nanonetwork SiO_2_ was applied as a protective layer for ITO conductive lines. Under compressive loading, unprotected ITO lines failed at 9 kgf, while protected lines endured up to 16.8 kgf with stable electrical resistance of 10.7 kΩ ± 3%, confirming the effectiveness of the coating in preventing mechanical damage. This work establishes a scalable route for fabricating multifunctional nanonetwork metamaterials, enabling their integration into protective overlayers, thin-film encapsulation, and stress-buffering interlayers for advanced semiconductors and photonic devices.

## 4 Experimental Section

### 4.1 Sample Preparation

A thin-film sample was prepared by spin coating. PS-*b*-PDMS was dissolved in cyclohexane, and the polymer solution was spin-coated at 2000 rpm for 60 s to form a PS-*b*-PDMS film on a quartz substrate. For each experimental condition, multiple independent samples were prepared to ensure reproducibility. Thin films for optical and mechanical characterization were primarily fabricated on 2 cm × 2 cm quartz substrates, while silicon or silica wafers were used for chemical and structural analyses. To drive the self-assembly of the PS-*b*-PDMS thin film into a gyroid structure, the film was placed into a custom-built solvent annealing chamber. The solvent annealing was conducted at ambient conditions using chlorobenzene as the selective solvent, with a solvent to nitrogen ratio of 50:3 for a duration of 2 h. Subsequently, a rapid solvent evaporation process was applied by abruptly terminating the solvent vapor supply and switching to pure nitrogen flow (solvent to nitrogen ratio of 0:100), effectively trapping the metastable gyroid morphology formed during solvent annealing. To observe the underlying structure, CF_4_/O_2_ RIE was employed to remove the top PDMS wetting layer. The RIE process was performed using a CF_4_/O_2_ gas mixture at a 2:1 ratio, with a power of 60 W and a pressure of 150 mTorr for 60 s. A gyroid-structured SiO_2_ thin film can be obtained through oxygen plasma treatment under controlled conditions: a power of 100 W and a flow rate of 10 sccm for 60 min. During this process, the PS template was etched away, yielding a nanoporous SiO_2_ thin film with a well-ordered network morphology.

### 4.2 Instrumentation

The atomic force microscopy (AFM) experiment was performed using a Seiko SPA-400 operating in tapping mode, with a SEIKO SPI-3800N station under ambient conditions. The Si pillar had an elastic constant of 15 N m^–1^ and a resonance frequency of 130 Hz. This setup allowed for detailed characterization of the sample surface geometry and properties. Field emission scanning electron microscopy (FESEM) was conducted using a HITACHI SU8010 with an accelerating voltage of 10 keV and a working distance of 4 mm. The samples were mounted on carbon tape and sputter-coated with platinum to prevent charging effects during observation. Small-angle X-ray scattering (SAXS) and grazing-incidence small-angle X-ray scattering (GISAXS) data were collected at the synchrotron X-ray beamline BL 23A at the National Synchrotron Radiation Research Center (NSRRC). The X-ray beam used had an energy of 15 keV and a wavelength of 0.155 nm. The 2D patterns were collected using a MAR CCD X-ray detector and subsequently converted into 1D linear profiles. An incident angle of 0.2 degrees was used for all GISAXS samples.

### 4.3 Mechanical Properties Measurement

Nanoindentation and nanoDMA measurements were performed on two SiO_2_ specimens using a Hysitron TI 980 TriboIndenter equipped with a 200 nm-radius Berkovich probe and calibrated following the Oliver–Pharr procedure. Nanoindentation was conducted under force control to a maximum load of 500 μN (100 μN s^–1^ loading/unloading; 5 s hold), while nanoDMA was carried out with an 18.22–18.26 nm displacement amplitude at 200 Hz for 1,394 cycles; additional compressive tests using an MTS system (0.45 mm tip) and resistance monitoring by a Keithley instrument were employed to assess the protective function of the nanonetwork SiO_2_ on ITO conductive lines.

### 4.4 Optical Properties Measurement

To examine the optical properties of the well-ordered nanonetwork silica thin film, UV–visible spectroscopy was performed using a USB4000 Fiber Optic Spectrometer from Ocean Optics. The light source used was a DH-2000-BAL Deuterium Tungsten Source, which provided a UV–vis-NIR wavelength range of 210 to 2500 nm. Both the transmittance and refractive index of the film were measured. UV–vis experiment was conducted over a wavelength range of 400–700 nm with a slit width of 1 nm to ensure precise light intensity reception. Air was used as the transmission reference baseline during the analysis.

## Supplementary Material



## Notes

Experimental procedures for the synthesis of PS-*b*-PDMS; characterization of self-assembled morphologies after solution casting using a neutral solvent (Figure S1) and a PS-selective solvent (Figure S2); schematic and setup of the solvent vapor annealing system (Figure S3); swelling behavior of PS-*b*-PDMS thin films during solvent annealing (Figure S4); GISAXS patterns at various solvent annealing times (Figure S5); top-view FESEM image of gyroid-structured PS-*b*-PDMS (Figure S6); cross-sectional FESEM image showing incomplete etching after RIE treatment (Figure S7); cross-sectional SEM images of gyroid-structured SiO_2_ films after oxygen plasma treatment. (Figure S8); GISAXS analysis of oxidized PS-*b*-PDMS films (Figure S9); chemical composition analysis by XPS and depth profiling (Figure S10); nanoindentation load–displacement curves of gyroid-structured SiO_2_ (Figure S11); mechanical damping behavior and impact-resistance comparison via nanoDMA (Figure S12); mechanical protection test of gyroid-structured SiO_2_ on ITO conductive lines (Figure S13); and optical transmittance of nanonetwork SiO_2_ thin films with different thicknesses (Figure S14). (PDF)
