# Supplementary material for: Metamaterials from Plasma-Treated Block Copolymer Monolith
Source: ACS Appl Mater Interfaces. 2026 Mar 23;18(13):19434–46. doi: 10.1021/acsami.5c25786 (PMC13067270; doi:10.1021/acsami.5c25786)
Supplement: Supplementary file 1 [file am5c25786_si_001.pdf]

Supporting Information for

Metamaterials from Plasma-Treated Block Copolymer Monolith

Chien Chen,[1] Jui-Chang Chuang,[2] Ke-Hsin Yin,[1] Cheng-Hsun Tung,[1] Kai-Cheng Yang,[3] Yu-Chueh Hung,[3] Chang-Chun Lee,[2] Rong-Ming Ho[1*]

[1] Department of Chemical Engineering, National Tsing Hua University No. 101, Section 2, Kuang-Fu Road, Hsinchu, Taiwan 30013, R.O.C.

[2] Department of Power Mechanical Engineering, National Tsing Hua University No. 101, Section 2, Kuang-Fu Road, Hsinchu, Taiwan 30013, R.O.C.

[3] Institute of Photonics Technologies, National Tsing Hua University No. 101, Section 2, Kuang-Fu Road, Hsinchu, Taiwan 30013, R.O.C.

* E-mail of corresponding author

rmho@mx.nthu.edu.tw

Synthesis of PS-*b*-PDMS

The synthesis of lamellae-forming PS-*b*-PDMS block copolymers involved anionic polymerization, utilizing a two-step sequence under high vacuum conditions. Initially, we prepared a sample with polystyrene (PS) and polydimethylsiloxane (PDMS) molecular weights of 12,800 g mol^{-1} and 10,400 g mol^{-1}, respectively, achieving a PDMS block volume fraction of 0.46. This process utilized sec-BuLi as the initiator and trimethylchlorosilane as the termination agent, with benzene and tetrahydrofuran as solvents to ensure purity and reactivity. **Table S1** presents the detailed characterization of the PS-*b*-PDMS samples used in this study.

Table S1. Characterization of the synthesized PS-*b*-PDMS.

**Sample**	M_n^{PS} **(kg mol⁻¹)** [a]	M_n^{PDMS} **(kg mol⁻¹)** [a]	**Đ** [b]	f_{PDMS}^{v} [c]
**PS-*b*-PDMS**	12.8	10.4	1.06	0.46

[a] Number-average molecular weights of two individual blocks (PS and PDMS) determined by membrane osmometry (MO).

[b] Polydispersity measured by size exclusion chromatography (SEC).

[c] Volume fraction of PDMS as calculated from proton nuclear magnetic resonance spectroscopy (1H-NMR) (ρ^{PS} = 1.04 g cm-3, ρ^{PDMS} = 0.97 g cm-3).

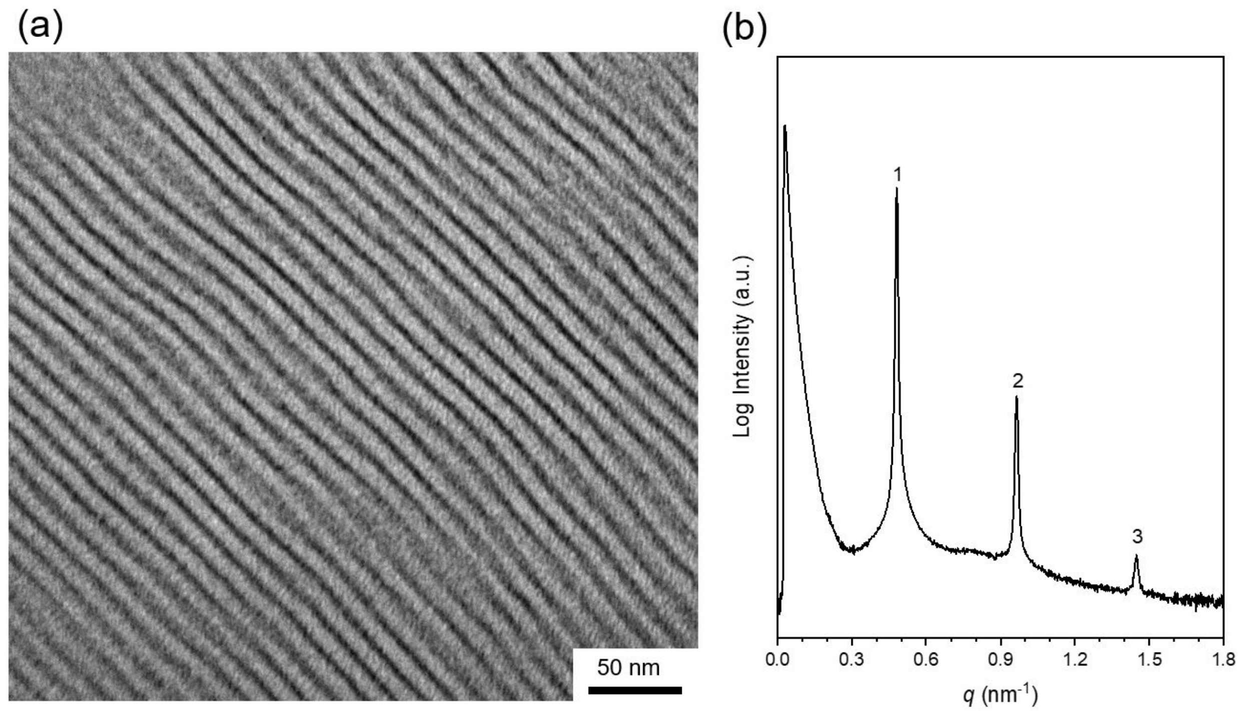

Figure S1. (a) TEM micrograph; (b) 1D SAXS profile of PS-*b*-PDMS after solution casting using a neutral solvent, cyclohexane.

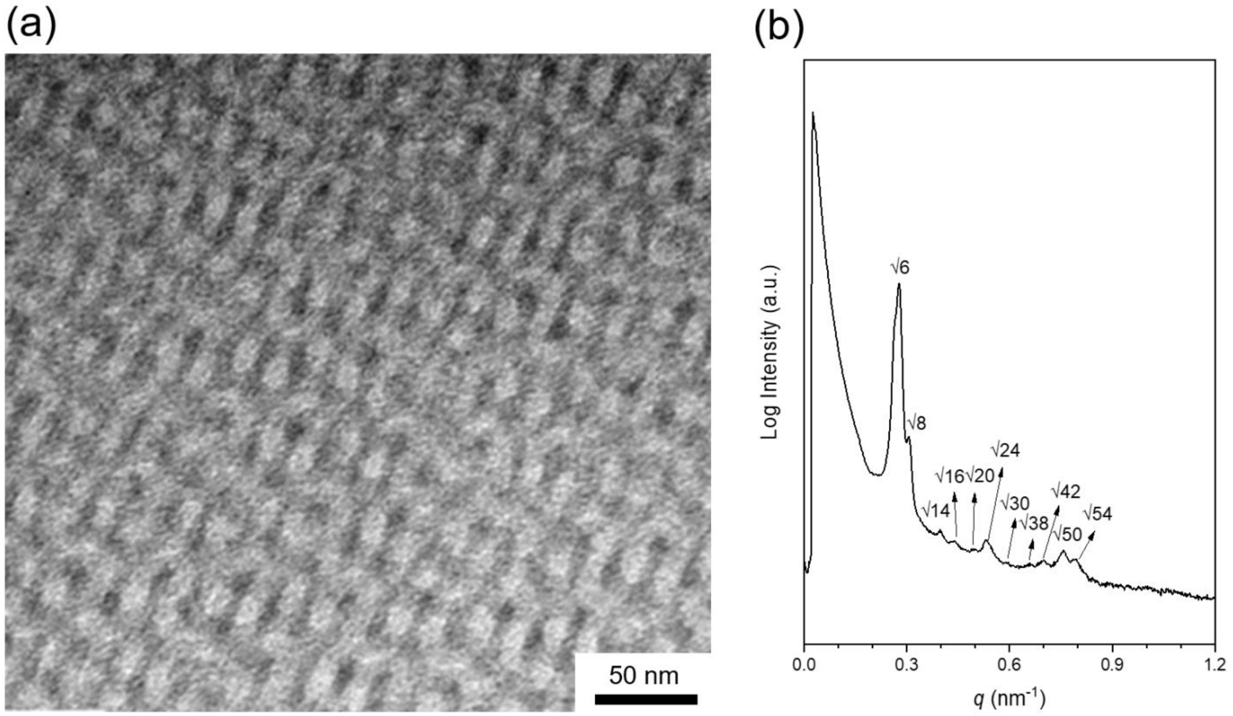

Figure S2. (a) TEM micrograph; (b) 1D SAXS profile of PS-*b*-PDMS after solution casting using a PS-selective solvent, chlorobenzene.

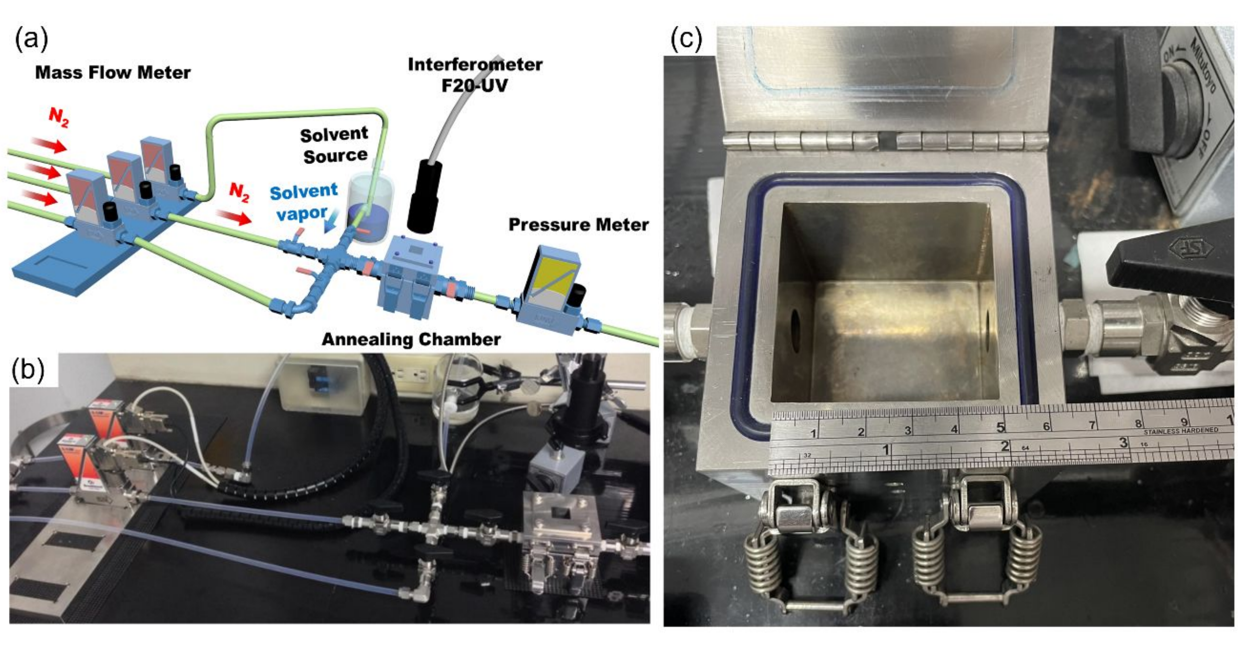

Figure S3. (a) Schematic illustration of the custom-built solvent vapor annealing (SVA) system, where nitrogen gas is mixed with solvent vapor and introduced into the annealing chamber under controlled flow rate and pressure. (b) Photograph of the experimental SVA setup. (c) Photograph of the sealed stainless-steel annealing chamber with an internal area of approximately 5 cm × 5 cm, used for thin-film annealing.

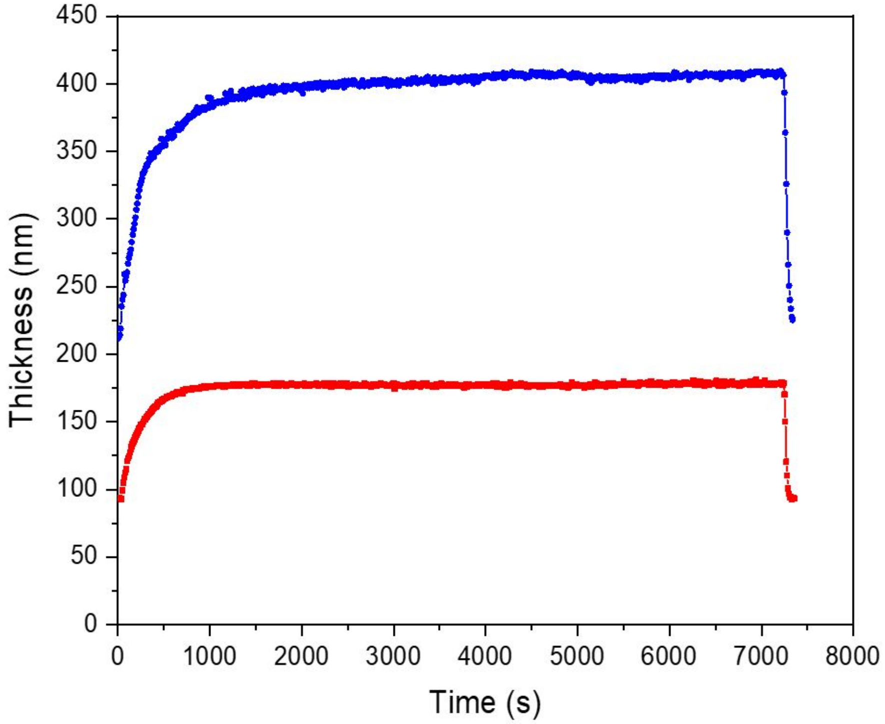

Figure S4. Swelling profiles of PS-*b*-PDMS thin films with different initial thicknesses during solvent annealing in chlorobenzene vapor, using a nitrogen carrier gas flow rate of 50 mL min^{-1} and chlorobenzene vapor flow rate of 3 mL min^{-1}. The thickness was monitored over 2 hours. The blue and red curves correspond to films with higher and lower initial thicknesses, respectively.

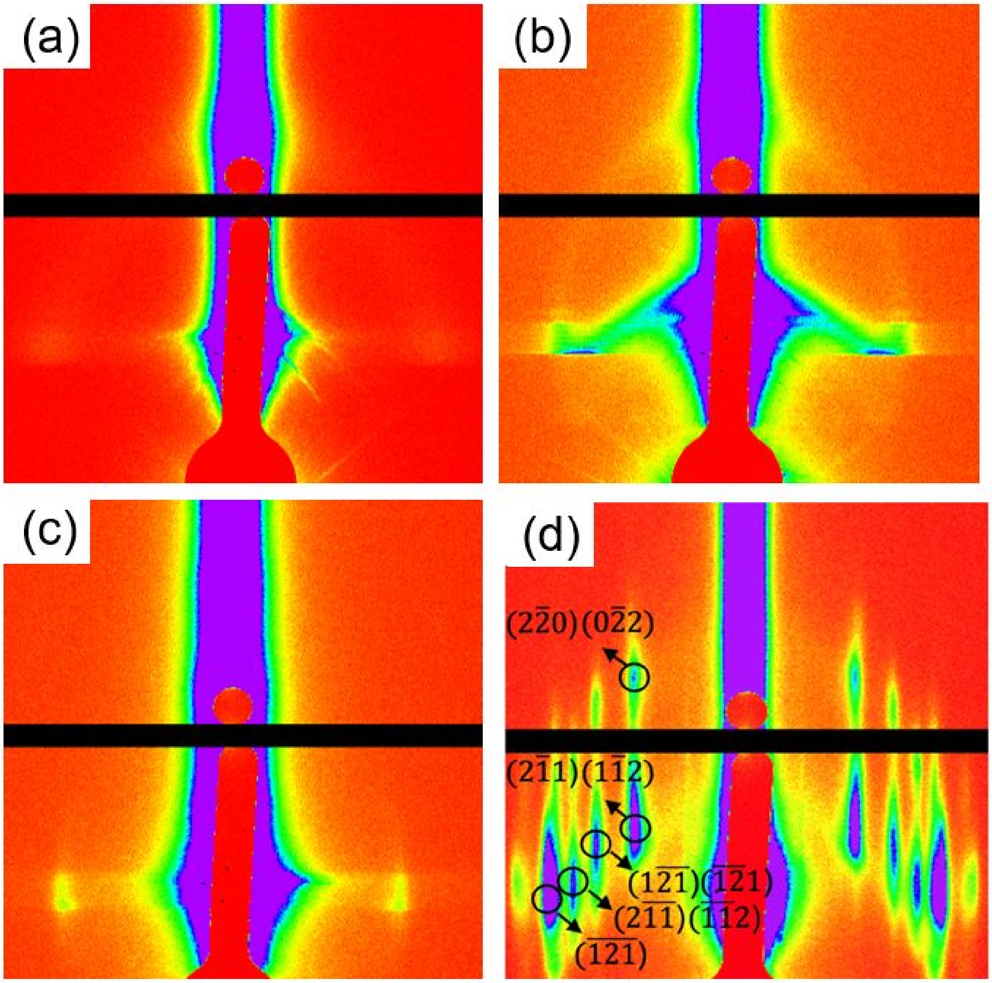

Figure S5. 2D GISAXS patterns of (a) as-cast PS-*b*-PDMS film and films after solvent annealing for (b) 5 minutes, (c) 30 minutes, and (d) 2 hours.

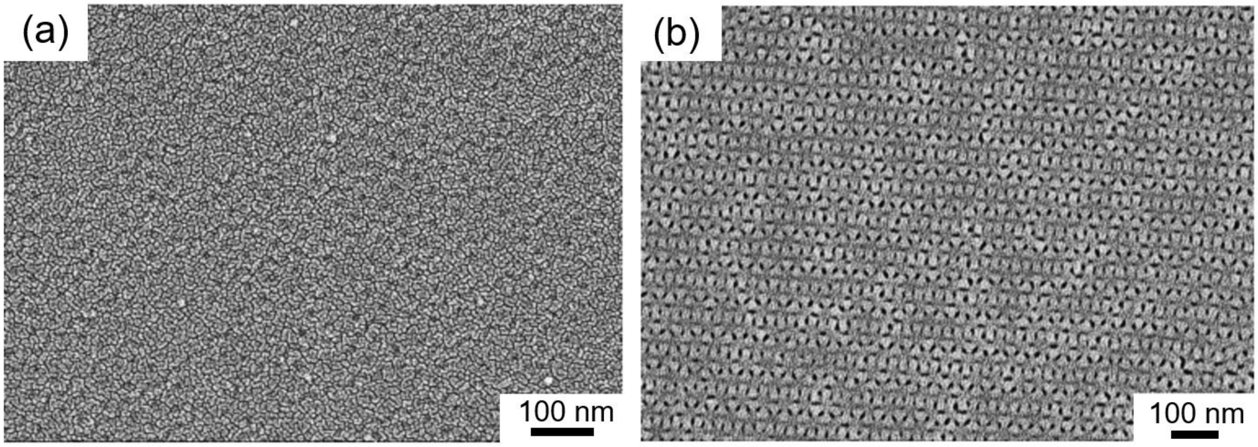

Figure S6. Top-view FESEM micrographs of gyroid-structured PS-*b*-PDMS thin film (a) without; (d) with removal of PDMS wetting layer by CF_4/O_2 RIE treatment followed by O_2 plasma treatment.

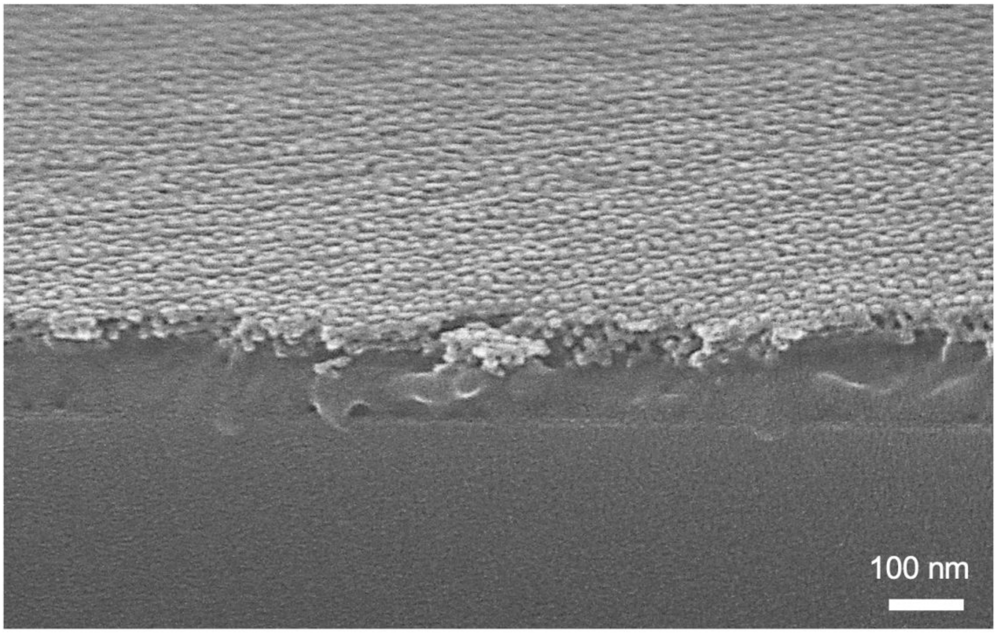

Figure S7. Cross-sectional SEM image of the sample after RIE treatment (100 W, 60 minutes), showing incomplete etching of the gyroid structure.

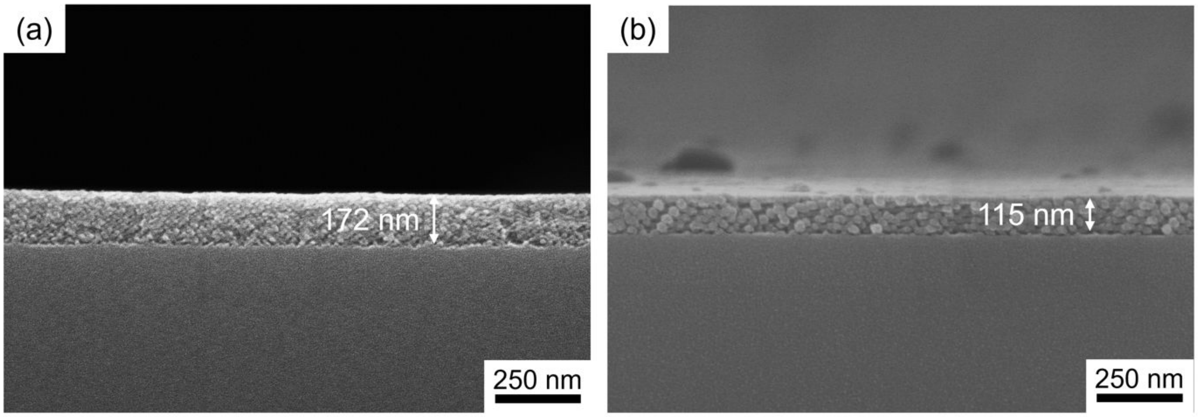

Figure S8. Cross-sectional SEM images of gyroid-structured SiO_2 films after oxygen plasma treatment. (a) Fully converted gyroid-structured SiO_2 film obtained under the standard plasma conditions. (b) Gyroid-structured SiO_2 film after an additional oxygen plasma treatment at 100 W for 60 minutes, showing a reduced film thickness, indicating slow etching of the amorphous SiO_2 upon prolonged plasma exposure.

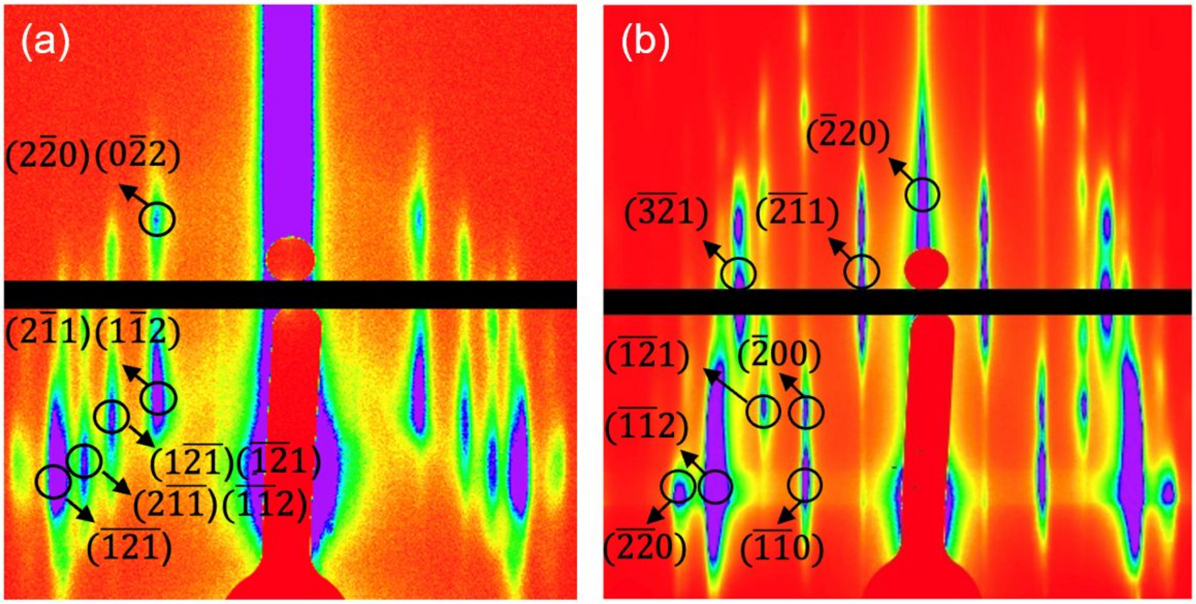

Figure S9. 2D GISAXS pattern of (a) PS-*b*-PDMS thin-film after solvent annealing; (b) Oxidized PS-*b*-PDMS thin film after oxygen plasma treatment.

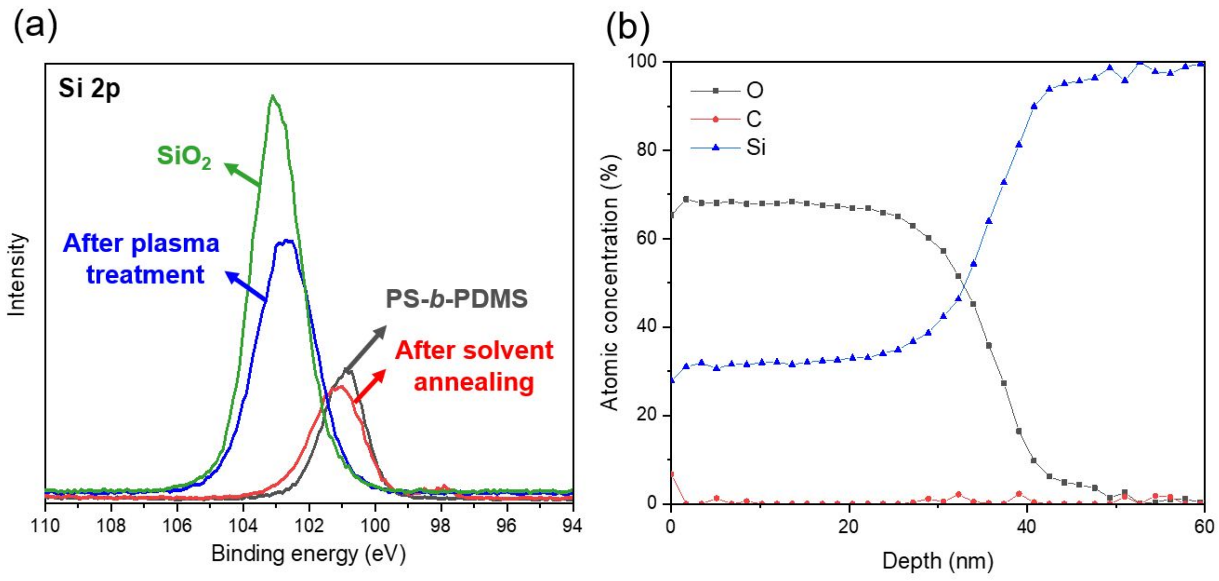

Figure S10. (a) HRXPS Si 2p spectra of PS-*b*-PDMS thin film before and after oxygen plasma treatment with a pure SiO_2 wafer as a reference. (b) XPS depth analysis of oxidized PS-*b*-PDMS thin film after oxygen plasma treatment.

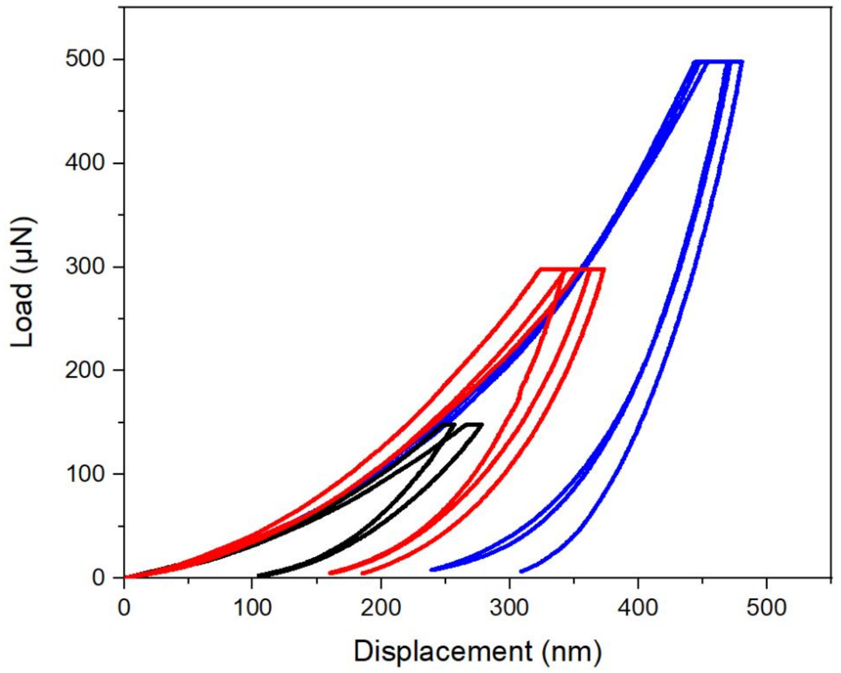

Figure S11. Represented load-displacement curves from nanoindentation multi-cycle tests on Gyroid-structured SiO_2 under three different peak loads (150, 300, and 500 μN).

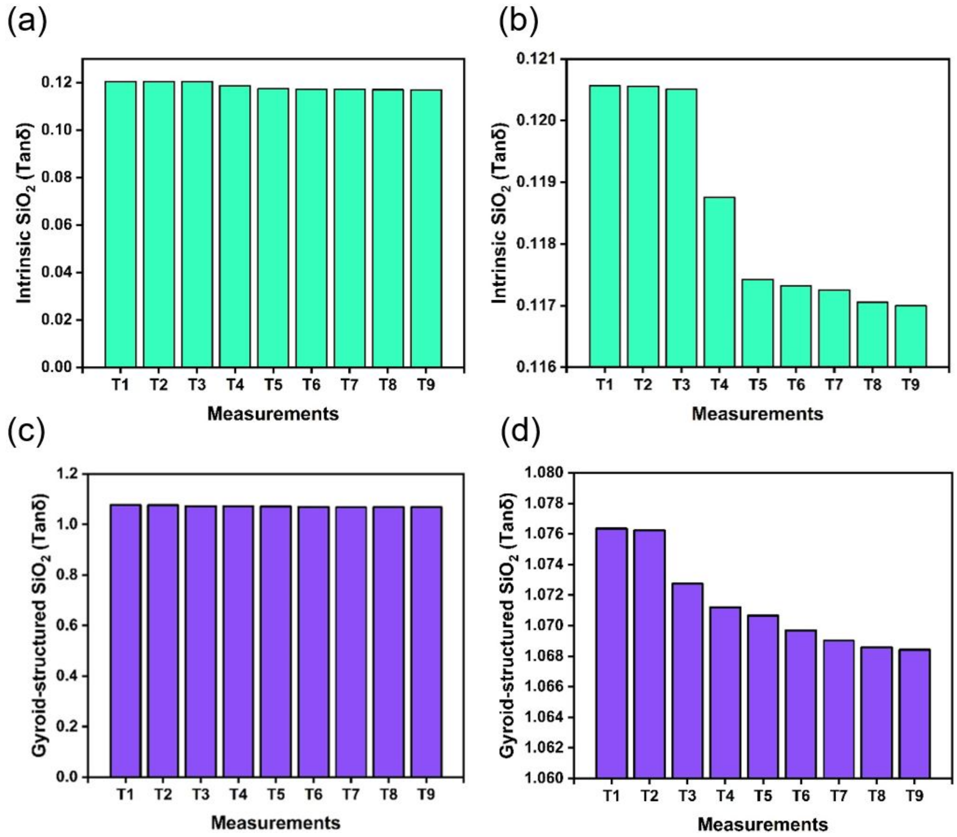

Figure S12. Impact resistance comparison between gyroid-structured SiO_2 and intrinsic SiO_2 *via* tan δ value from nanoDMA. (a) tan δ value for intrinsic SiO_2. (b) Magnified view of impact resistance changes for intrinsic SiO_2. (c) tan δ value for the gyroid-structured SiO_2. (d) Magnified view of impact resistance changes for the gyroid-structured SiO_2.

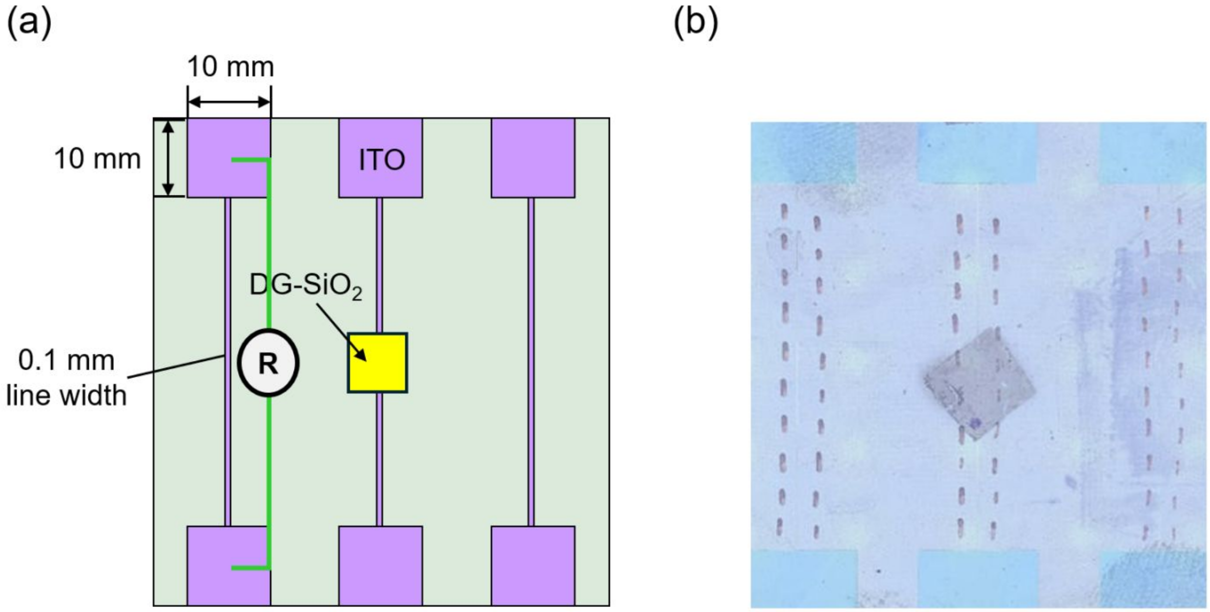

Figure S13. (a) Schematic illustration of the experimental setup for evaluating the mechanical protection performance of gyroid-structured SiO_2 on ITO conductive lines. The test device comprises parallel ITO lines (width: 0.1 mm) patterned on a substrate with 10 mm spacing. A gyroid-structured SiO_2 thin film (yellow square) is selectively placed on top of the central ITO line, which is electrically connected to a resistance measurement system. Under compressive loading applied *via* a mechanical test system, the change in resistance is monitored to assess the mechanical integrity of the ITO lines with and without gyroid-structured SiO_2 protection. (b) Optical microscopy image of the fabricated device, showing the patterned ITO lines and the centrally positioned gyroid-structured SiO_2 thin film.

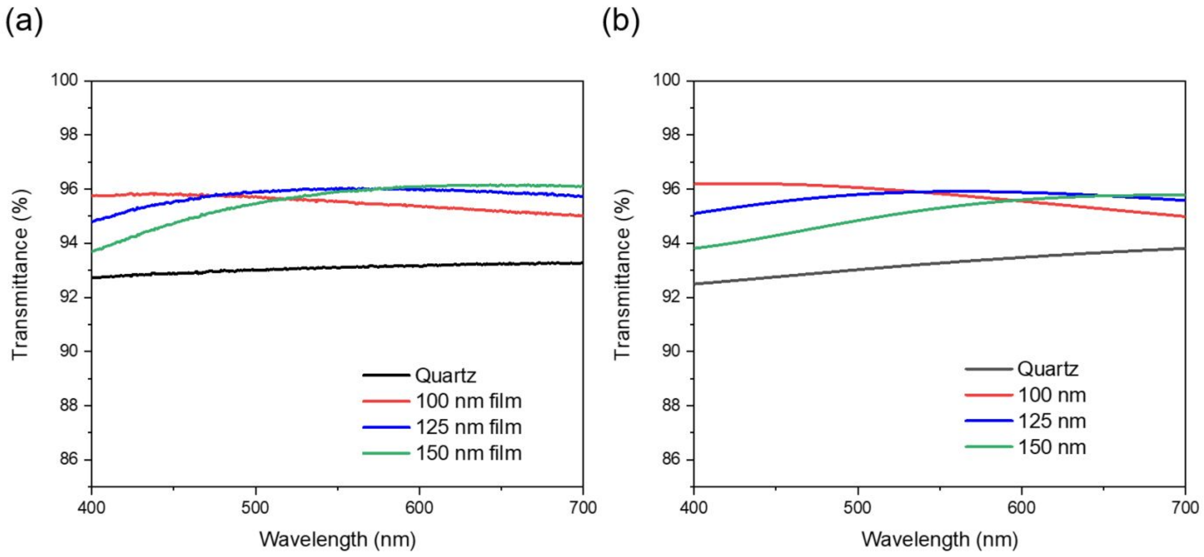

Figure S14. Optical transmittance of gyroid-structured SiO_2 films with varying thicknesses (100 nm, 125 nm, and 150 nm) on quartz substrates: (a) experimental and (b) simulated results in the wavelength range of 400-700 nm.
